# Syndecan-2 is upregulated in colorectal cancer cells through interactions with extracellular matrix produced by stromal fibroblasts

**DOI:** 10.1186/1471-2121-14-25

**Published:** 2013-05-25

**Authors:** Carolina Meloni Vicente, Ritchelli Ricci, Helena Bonciani Nader, Leny Toma

**Affiliations:** 1 Disciplina de Biologia Molecular, Departamento de Bioquímica, Universidade Federal de São Paulo, UNIFESP, Rua Três de Maio, 100 – 4º andar, Vila Clementino, São Paulo, SP CEP 04044-020, Brazil

**Keywords:** Colorectal cancer, Cancer-stroma interaction, Proteoglycans, Syndecan-2, Fibronectin

## Abstract

**Background:**

The extracellular matrix (ECM) influences the structure, viability and functions of cells and tissues. Recent evidence indicates that tumor cells and stromal cells interact through direct cell-cell contact, the production of ECM components and the secretion of growth factors. Syndecans are a family of transmembrane heparan sulfate proteoglycans that are involved in cell adhesion, motility, proliferation and differentiation. Syndecan-2 has been found to be highly expressed in colorectal cancer cell lines and appears to be critical for cancer cell behavior. We have examined the effect of stromal fibroblast-produced ECM on the production of proteoglycans by colorectal cancer cell lines.

**Results:**

Our results showed that in a highly metastatic colorectal cancer cell line, HCT-116, syndecan-2 expression is enhanced by fibroblast ECM, while the expression of other syndecans decreased. Of the various components of the stromal ECM, fibronectin was the most important in stimulating the increase in syndecan-2 expression. The co-localization of syndecan-2 and fibronectin suggests that these two molecules are involved in the adhesion of HCT-116 cells to the ECM. Additionally, we demonstrated an increase in the expression of integrins alpha-2 and beta-1, in addition to an increase in the expression of phospho-FAK in the presence of fibroblast ECM. Furthermore, blocking syndecan-2 with a specific antibody resulted in a decrease in cell adhesion, migration, and organization of actin filaments.

**Conclusions:**

Overall, these results show that interactions between cancer cells and stromal ECM proteins induce significant changes in the behavior of cancer cells. In particular, a shift from the expression of anti-tumorigenic syndecans to the tumorigenic syndecan-2 may have implications in the migratory behavior of highly metastatic tumor cells.

## Background

The extracellular matrix (ECM) is an extremely complex supramolecular structure that is locally secreted by cells and influences the structure, viability and functions of cells and tissues. Epithelial-mesenchymal and epithelial-stromal interactions play important roles in both physiological and pathological settings, such as during embryonic morphogenesis [1,2], wound healing [3] and tumorigenesis [4], and are accompanied by dynamic changes that generate new cell-matrix interactions [5]. Cell contact with a single ECM component initiates multiple signals that affect both cell behavior and gene expression [6,7].

The tumor microenvironment plays an important role in the behavior of malignant cells. The functional association of cancer cells with surrounding tissues changes as the malignancy progresses and is accompanied by the remodeling of the ECM [8]. A severe fibrotic reaction around tumor tissues represents a host reaction to tumor growth. Fibroblasts are the main source of fibrotic tissue, which consists mainly of collagen types I and IV. In addition to collagen, several other ECM proteins are present, including fibronectin, laminin, tenascin and proteoglycans [9]. Increasing evidence has demonstrated the involvement of cell surface proteoglycans in the multi-functional network of cell-cell and cell-matrix interactions that are essential for local tumor invasion and subsequent metastasis [10-13].

Syndecan-2 is one of the four members of the syndecan family of cell surface transmembrane heparan sulfate (HS) proteoglycans. It has been implicated in the formation of specialized membrane domains and functions as a direct link between the extracellular environment and the organization of the cortical cytoplasm. In several colorectal cancer cell lines, syndecan-2 is highly expressed compared to normal cell lines [14-16]. This increase appears to be critical for cancer cell behavior because it regulates adhesion and proliferation and, therefore, tumorigenic activity [17]. In order to examine the relationship between tumor cells and stromal fibroblasts, we used an *in vitro* model to investigate the effect of ECM that is produced by stromal fibroblasts on the synthesis of glycosaminoglycans (GAGs) and proteoglycans by two colorectal cancer cell lines, Caco-2 and HCT-116, which have different metastatic potentials.

## Results

### Stromal fibroblast ECM influences GAG synthesis by HCT-116 colorectal cancer cells

To analyze the interactions between tumor cells and stromal fibroblast ECM, two colorectal cancer cell lines with different metastatic potentials, Caco-2 and HCT-116 cells, were studied. The influence of stromal fibroblast-produced ECM on GAG and proteoglycan synthesis by the cancer cells was investigated.

Tumor cells were cultured for three days on top of stromal ECM and then labeled with [^35^S]Na_2_SO_4_. The GAGs synthesized were analyzed by agarose gel electrophoresis in 0.05-M 1,3-diaminepropane acetate buffer and quantified as described in the Methods.

**Figure 1:**
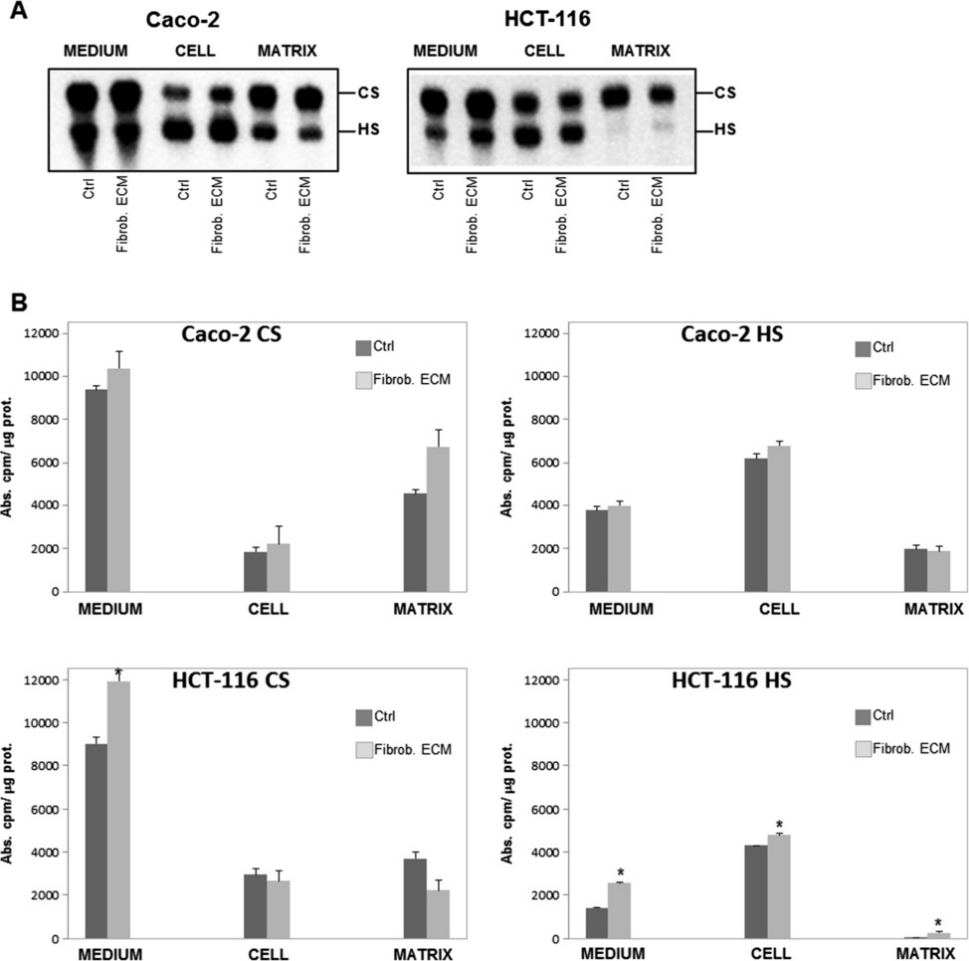
**Effect of stromal fibroblast ECM on the synthesis of GAGs by Caco-2 and HCT-116 cells.** Cancer cells were cultured in the absence (Ctrl) or presence (Fibrob. ECM) of stromal fibroblast ECM. GAGs were labeled with [^35^S]Na_2_SO_4_ and were purified from the culture medium (MEDIUM), cancer cells (CELL) and the matrix (MATRIX) produced by Caco-2 or HCT-116 cells. (**A**) The content of GAGs from these compartments was analyzed by agarose gel electrophoresis in 1,3-diaminepropane acetate buffer (0.05-M pH 9.0). The gel was exposed to a screen and the bands were identified using an image analysis system, the Cyclone® Storage Phosphor System-Packard Instrument. (**B**) Quantification was performed by densitometry with Opti-Quanti Software. Heparan sulfate (HS), chondroitin sulfate (CS). *p ≤ 0.05 compared to control.

The Caco-2 colorectal cancer cell line, which has low metastatic potential, produces both chondroitin sulfate (CS) and HS, the former being more abundant in the medium and the latter being more abundant in cell extracts (Figure 1). There was no difference in GAG synthesis by Caco-2 cells in the presence or absence of stromal fibroblast ECM. In the HCT-116 colorectal cell line, which has high metastatic potential, the same GAG distribution profile was observed, but there was a significant increase in medium, cell extract and matrix HS production when cells were cultured on top of stromal ECM (Figure 1).

### Gene expression of surface and ECM proteoglycans is modulated by stromal fibroblast ECM

Proteoglycan expression was analyzed to confirm the GAG synthesis results. We investigated the expression of the family of syndecans (-1, -2, -3 and -4), which have been shown to be involved in cancer-stroma interactions [15,18].

**Figure 2:**
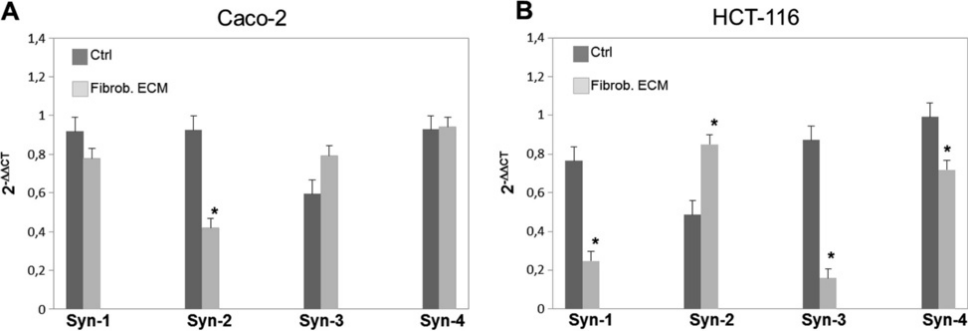
**Effect of stromal fibroblast ECM on the expression of syndecans in Caco-2 and HCT-116 cells.** Caco-2 (**A**) and HCT-116 cells (**B**) were cultured on Petri dishes in the absence (Ctrl) or presence of fibroblast ECM (Fibrob. ECM) for three days, and RNA was extracted. The expression level of each gene was normalized to that of β-actin. The data from each experiment were obtained in triplicate and are represented as the average ± standard deviation. *p ≤ 0.05. Syndecan-1 (Syn-1), syndecan-2 (Syn-2), syndecan-3 (Syn-3), syndecan-4 (Syn-4).

Stromal fibroblast ECM did not significantly affect the expression of syndecans in Caco-2 cells, with the exception of syndecan-2, which decreased in the presence of stromal fibroblast ECM (Figure 2). Many colon cancer cell lines show increased syndecan-2 expression, and this up-regulation seems to be crucial for their tumorigenic activity. In contrast, colon cancer cell lines HT29, Caco-2 and DLD1 show low syndecan-2 expression, and inhibition of syndecan-2 function in these cell lines did not affect any of their adhesion, proliferation, invasion and migration [15].

In opposition, stromal fibroblast ECM decreased syndecan-1, -3 and -4 expression, but significantly increased syndecan-2 expression in HCT-116 cells (Figure 2). Of the four syndecan family members, syndecan-2 has been previously reported as up-regulated in several colon carcinoma cells and, further, that this increased expression is crucial for tumorigenicity [19,20].

### Stromal fibroblast ECM influences the synthesis of syndecan-2 by HCT-116 colorectal cancer cells

**Figure 3:**
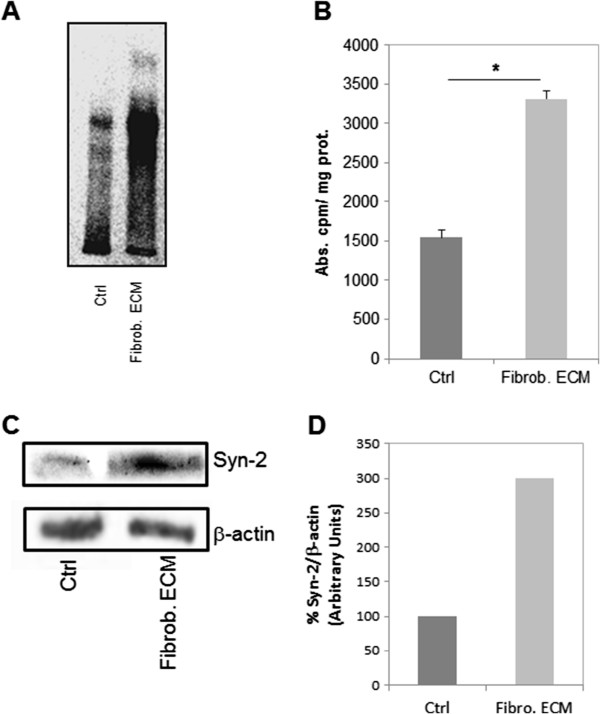
**Expression of syndecan-2 in HCT-116 cells.** (**A**) Immunoprecipitation of syndecan-2 from HCT-116 cells in the absence of matrix (Ctrl) or in the presence of stromal fibroblast ECM (Fibrob. ECM). HCT-116 cells were cultured for 72h and exposed to [35S]sulfate for 24 h, and the radioactive proteoglycans were extracted as described in Methods. The proteoglycans from the cells were immunoprecipitated with anti-syndecan-2 antibody and were then applied to the gel. (**B**) Quantification of the experiment shown in A. (**C**) HCT-116 cells were seeded on stromal fibroblast ECM and cultured for three days. Lysate proteins were separated on 10% SDS-PAGE and electro-transferred to PVDF membrane. Membranes were blocked and incubated using anti-syndecan-2 (Syn-2) and anti-β-actin (loading control). Antibody binding was visualized by chemiluminescence and the relative levels of these proteins were determined by densitometric analysis (**D**). *p ≤ 0.05 compared to control.

To confirm the increase in HCT-116 syndecan-2 expression that was induced by stromal fibroblast ECM at the protein level, and to relate it with GAGs synthesis, the cells were labeled with [^35^S]sulfate and immunoprecipitated with an antibody against the extracellular domain of human syndecan-2 proteoglycan (Figure 3A). The results clearly indicate that the stromal fibroblast increases the amount of syndecan-2 in HCT-116 cells (Figure 3B). Furthermore, the results show the association of HS augments with this syndecan isoform. Through western blotting, a 150% increase of syndecan-2 in HCT-116 cells cultured in the presence of stromal fibroblasts ECM was observed therefore corroborating the immunoprecipitation (Figure 3C and D).

**Figure 4:**
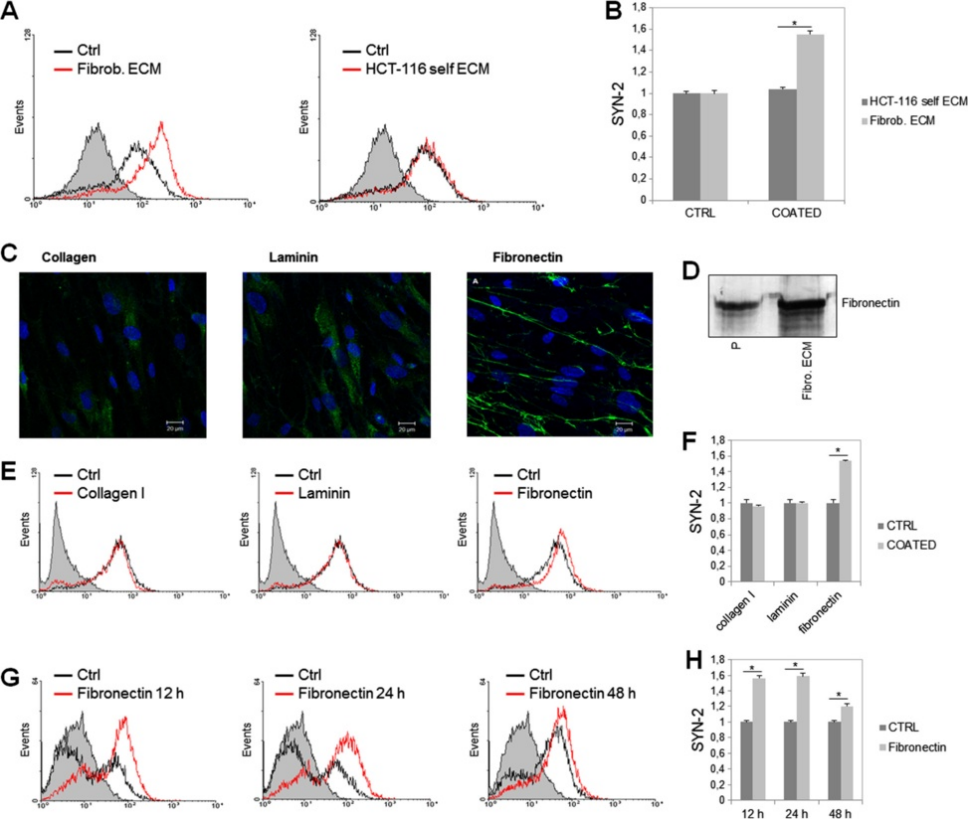
**Flow cytometric analysis of syndecan-2 surface expression on HCT-116 cells.** (**A**) HCT-116 cells were cultured for 48 h in the absence of matrix (Ctrl), in the presence of stromal fibroblast ECM (Fibrob. ECM) or in the presence of their own matrix (HCT-116 self ECM) and then immunostained with anti-syndecan-2 antibody. (**B**) The relative staining was determined by densitometric analysis. (**C**) Fibroblasts were immunostained with anti-fibronectin antibody, anti-laminin antibody or anti-collagen antibody to confirm the presence of these proteins. The photo shows images that were obtained using a confocal microscope. HCT-116 cells were cultured for 72 h on fibronectin, laminin or collagen I and labeled with anti-syndecan-2 antibody. (**D**) Fibroblasts were cultured in Petri dishes until confluence and its ECM was extracted as described in Methods. Total protein from the ECM (Fibro. ECM) was then applied to polyacrylamide gel 7.5% with 10μg of standard fibronectin (P). After transfer, the nitrocellulose membrane was incubated with anti-fibronectin and revealed with DAB. Collagen and laminin were not detected on ECM produced by fibroblasts through Western blotting. (**E**) HCT-116 cells were cultured in the presence of collagen-I, laminin or fibronectin for 72 h and then stained with anti-syndecan-2 antibody and the relative levels of this protein were determined by densitometric analysis (**F**). (**G**) HCT-116 cells were cultured in the absence or presence of fibronectin for different lengths of time (12, 24, 48 and 72 h) and then stained with anti-syndecan-2 antibody and the relative staining was determined by densitometric analysis (**H**). The gray peak represents the control cells cultured in the absence of ECM, the black line represents cells cultured in the presence of ECM and the gray line represents the control for the secondary antibody, anti-IgG. *p ≤ 0.05 compared to control.

We also detected cell surface syndecan-2 levels by flow cytometry. HCT-116 cells were cultured for three days in the presence or absence of stromal fibroblast ECM. As a control, cells were cultured in the presence of their own ECM. Significant levels of cell surface syndecan-2 were detected by flow cytometry when cells were grown on fibroblast ECM but not on their own ECM (Figure 4A). An increase in the expression of syndecan-2 as observed (Figure 4B).

We next investigated which specific component of the ECM was responsible for stimulating the increase in syndecan-2 expression. Initially, fibroblasts were immunostained with anti-fibronectin, anti-laminin or anti-collagen to confirm their presence in the ECM (Figure 4C). The expression of fibronectin was also confirmed through Western blotting, however collagen and laminin were not detected on ECM produced by fibroblasts through this method (Fgure 4D). Flow cytometry was then performed on HCT-116 cells grown in culture dishes coated with fibronectin, laminin or collagen type I for three days (Figure 4E). Laminin and collagen type I did not increase syndecan-2 expression on HCT-116 cells over control conditions. However, fibronectin stimulated an increase in syndecan-2 expression (Figure 4F).

To further investigate this fibronectin-induced increase in syndecan-2 expression, HCT-116 cells were cultured in the presence of fibronectin for different lengths of time (Figure 4G). Surprisingly, short incubation (12 h and 24 h) with fibronectin led to a remarkable increase in syndecan-2 expression (Figure 4H). Overall, our results show that stromal ECM induces the expression and synthesis of syndecan-2 in HCT-116 colorectal cancer cells and that the fibronectin component of ECM is mainly responsible for this effect.

**Figure 5:**
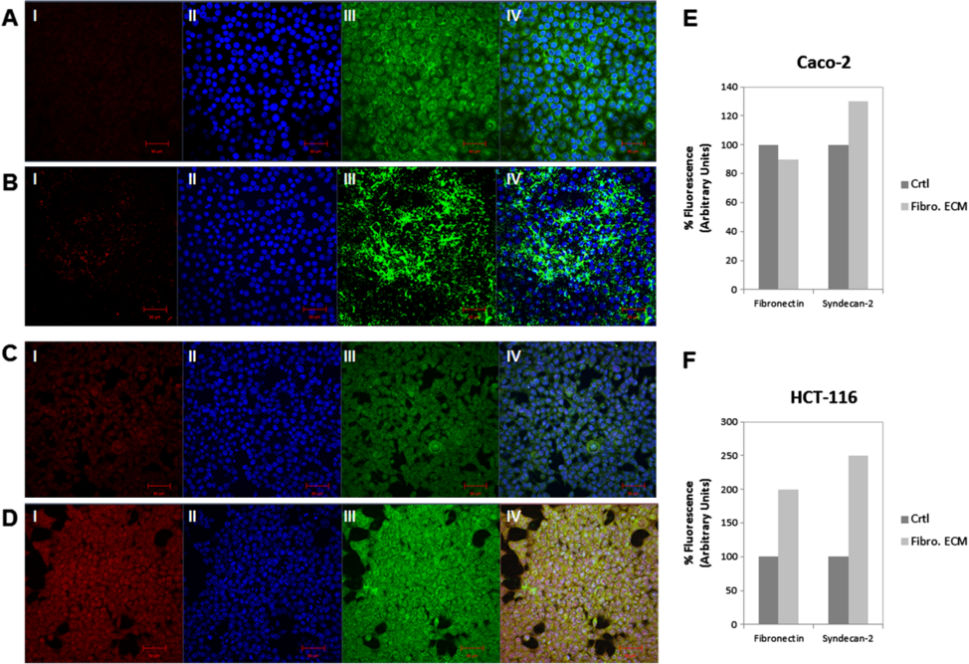
**Co-localization of syndecan-2 and fibronectin on Caco-2 and HCT-116 cells.** (**A**) Caco-2 cells were cultured on Petri dishes in the absence(Ctrl) or (**B**) presence of fibroblast ECM (Fibro. ECM) for three days. (**C**) HCT-116 cells were cultured on Petri dishes in the absence (Ctrl) or (**D**) presence of fibroblast ECM (Fibrob. ECM) for three days. Cells were immunestained with anti-fibronectin (III, green) and anti-syndecan-2 (I, red). The nuclei (II, blue) were stained with DAPI. Immunofluorescent images were captured using confocal microscopy. Co-localization of images (IV, Merge). Scale bar represents 20 μm. The relative fluorescence levels of proteins were determined by densiometric analysis and represented as a percentage of controls (**E** and **F**). *p ≤ 0.05 compared to control.

Taking into account the influence of fibronectin in the expression of syndecan-2, we analyzed the co-localization of these molecules through immunocytochemistry. Caco-2 cells presented no staining for syndecan-2, even in the presence of stromal fibroblasts ECM (Figure 5A and B), and there was a slight increase in the staining of fibronectin, probably due to protein produced by the fibroblasts (Figure 5E). On the other hand, a strong staining for syndecan-2 was observed at the HCT-116 cells, especially in the presence of stromal fibroblasts ECM (Figure 5C and F), and co-localized with fibronectin, which confirms our previous results (Figure 5D).

### Syndecan-2 participates in the cell adhesion, migration and organization of actin filaments in HCT-116 cells

**Figure 6:**
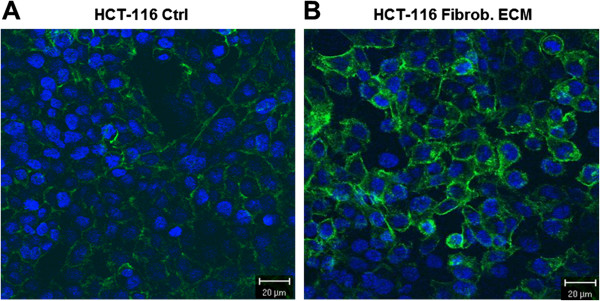
**Distribution of actin filaments in HCT-116 cells.** Cells were cultured in Petri dishes containing glass coverslips in the absence (**A**) or presence (**B**) of stromal fibroblast ECM. Actin filaments were stained with phalloidin conjugated to Alexa 488 (green). The cells were counterstained with DAPI to detect the nucleus (blue). The photo above shows images that were obtained using confocal microscopy. Scale mark: 20 μm.

Interactions between ECM molecules and cell surface receptors, such as heparan sulfate proteoglycans and integrins, can modulate the organization of the cytoskeleton. Therefore, we examined whether stromal fibroblast ECM regulated the organization of actin filaments in HCT-116 cells. Cells that were cultured on fibroblast ECM showed a higher organization of cortical actin filaments, indicating reorganization of the cytoskeleton (Figure 6).

**Figure 7:**
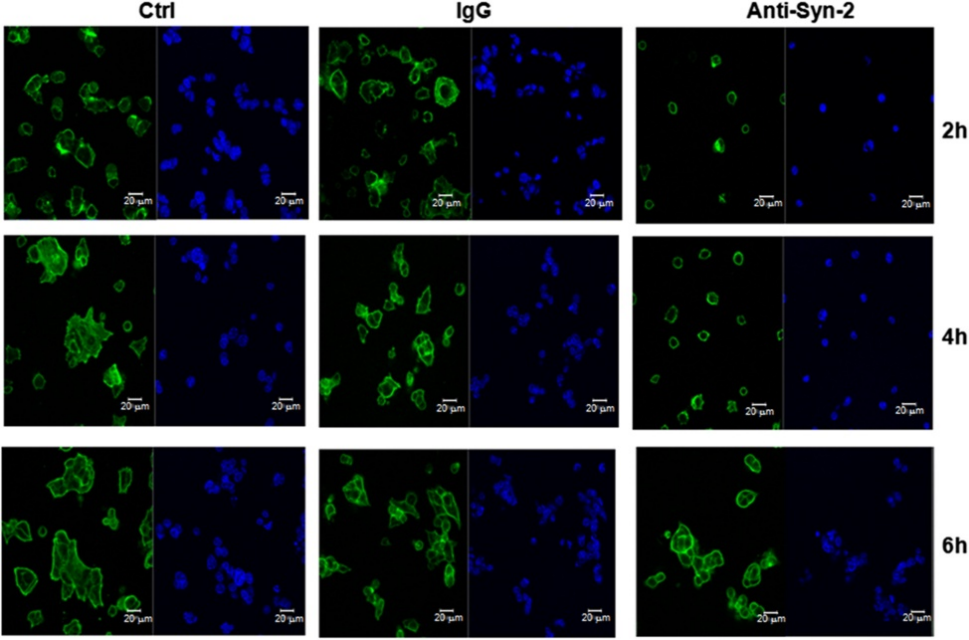
**Effect of syndecan-2 blockade on actin filament formation in HCT-116 cells.** HCT-116 cells, previously grown on top of fibroblast ECM for three days, were incubated with anti-syndecan-2 antibody (Anti-Syn-2), IgG (IgG) or no antibodies (Ctrl) and then plated on stromal fibroblast ECM-coated glass coverslips for different lengths of time. Actin filaments were stained. The image above was obtained by fluorescence microscopy. F-actin is labeled with phalloidin in green; nucleus is labeled with DAPI in blue. Scale mark: 20 μm.

**Figure 8:**
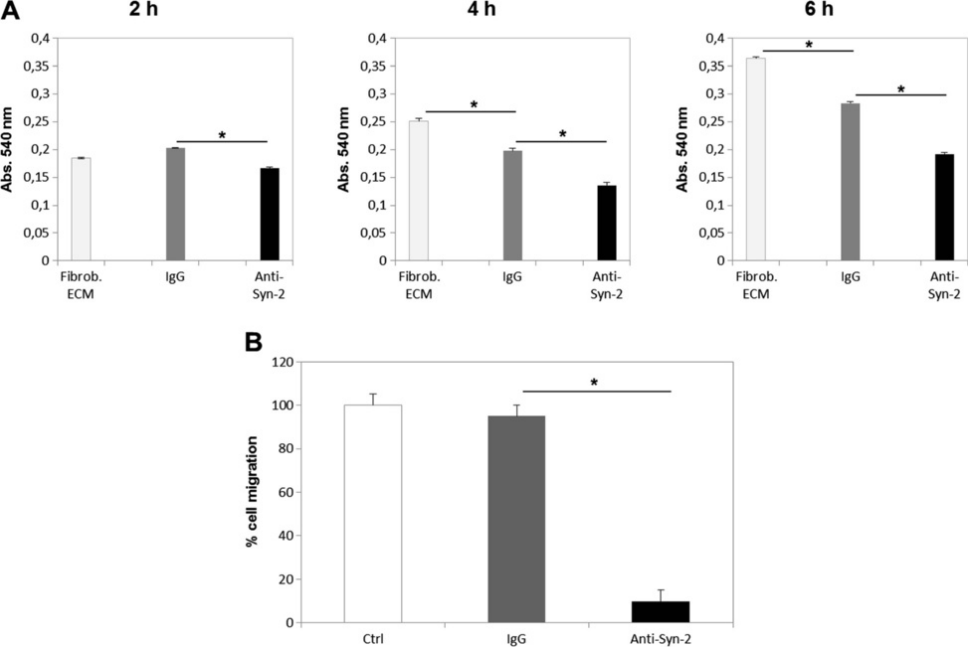
**Effect of syndecan-2 blockade on adhesion and migration of HCT-116 cells to stromal fibroblast ECM.** (**A**) HCT-116 cells, previously grown on top of fibroblast ECM for three days, were incubated with anti-syndecan-2 antibody (Anti-Syn-2), IgG (IgG) or no antibodies (Fibrob. ECM) and then plated on stromal ECM-coated Petri dishes for different lengths of time. Non-adherent cells were removed, and adhesion was measured by MTT colorimetric assay. The formazan crystals formed were solubilized with DMSO, and the absorbance was measured at 540 nm. (**B**) Transwell membranes (Costar, Corning, 8-μm pore size) were coated with stromal fibroblast ECM as described above. HCT-116 were pre-incubated with anti-syndecan-2 antibody (Anti-Syn-2), IgG (IgG) or no antibodies (Ctrl) and plated in the top of chamber. Migrating cells were fixed in formaldehyde, stained with crystal Violet, and counted. * *p ≤ 0.05.*

We next investigated whether syndecan-2 played a role in cell adhesion, migration and actin filament formation. HCT-116 cells, previously grown on top of fibroblasts ECM, were pre-incubated with an anti-syndecan-2 blocking antibody and then plated on stromal fibroblast ECM for different lengths of time. Cells that were pre-treated with anti-syndecan-2 remained isolated and did not aggregate like normal cells in this cell line. In control cells that were pre-incubated with control IgG or no antibodies, a higher organization of actin filaments was observed over time, as well as the formation of plasma membrane extensions that are indicative of stress fibers. Cells that were pre-treated with anti-syndecan-2 blocking antibody did not form stress fibers and remained round with cortical actin (Figure 7). Moreover, they were less adherent to the ECM (Figure 8A).

Previous studies have implicated syndecan-2 in HCT-116 cell motility [16,20]. To investigate this hypothesis, transmigration assays were performed. HCT-116 cells were pre-incubated with anti-syndecan-2, human IgG or no antibodies, seeded on transwell membranes coated with stromal fibroblast ECM and allowed to migrate for 24 h. Migratory cells on the bottom chamber of the transwell were counted with an inverted microscope. Cells in which syndecan-2 was blocked did not migrate through the transwell membrane (Figure 8B).

**Figure 9:**
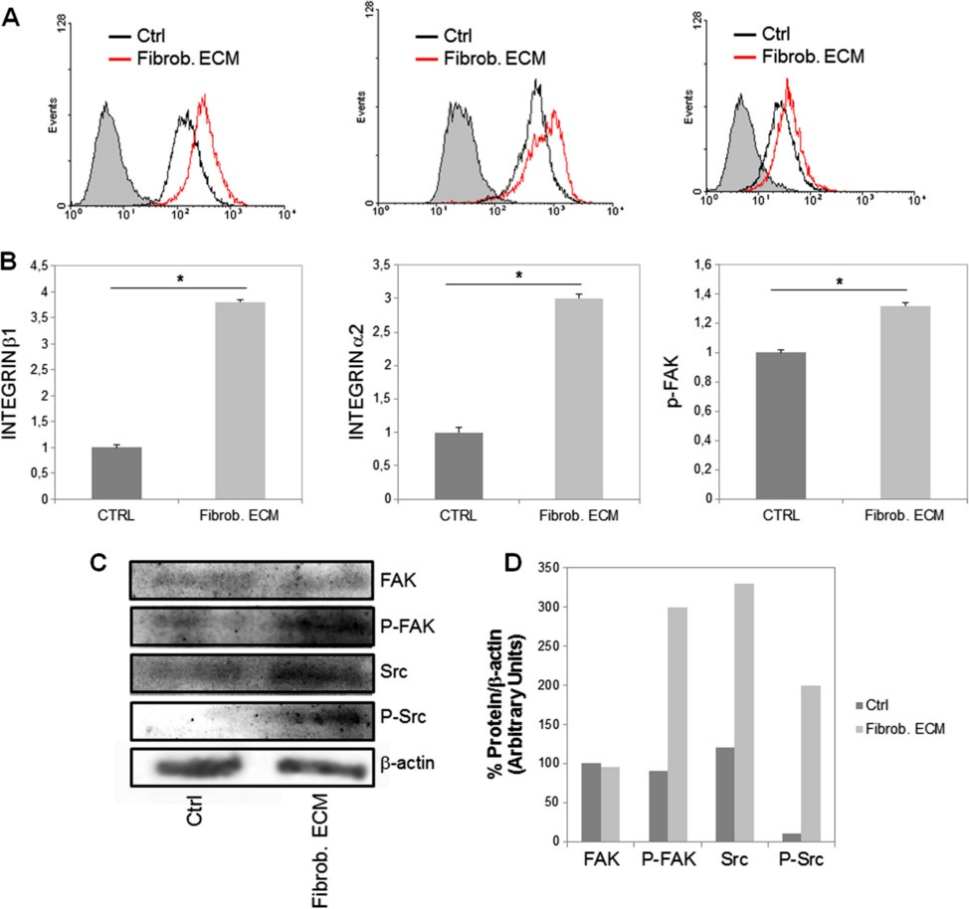
**Effect of stromal fibroblast ECM on the expression of integrins β1 and α2, and phosphorylation of FAK and Src in HCT-116 cells.** (**A**) HCT-116 cells were cultured for 48 h in the absence of matrix (Ctrl), in the presence of stromal fibroblast ECM (Fibrob. ECM) and then immunostained with anti-integrin-β1, anti-integrin-α2 or anti-phospho-FAK. The staining was analyzed through flow cytometry. (**B**) The relative staining was determined by densitometric analysis. (**C**) HCT-116 cells were seeded on stromal fibroblast ECM and cultured for 48 h. Lysate proteins were separated on 10% SDS-PAGE and electro-transferred to PVDF membrane. Membranes were blocked and incubated using anti-FAK, anti-phospho-FAK, anti-Src, anti-phospho-Src and anti-β-actin (loading control). Antibody binding was visualized by chemiluminescence and the relative levels of these proteins were determined by densitometric analysis (**D**). *p ≤ 0.05 compared to control.

To further investigate the mechanisms involved in the increment of cell adhesion, we analyzed the expression of integrins through flow cytometry. Or results showed a three fold increase in the expression of integrins alpha-2 and beta-1 (Figure 9A and B), in addition to an increase in the expression of phospho-FAK.

The increase in phosphorylation of FAK and Src has been correlated to metastatic potential of different cancers. Src family kinase and FAK activity are required for cell adhesion, spread and migration. Therefore, the signaling pathways were analyzed primarily through western blotting of cell extracts. Quantitative densitometry enabled the quantification of the levels of Src^Y416^, FAK^Y397^ as well as of their phosphorylated form, thereby, determining the level of activation of the signaling pathways. HCT-116 cells that were cultured on fibroblast ECM showed an increase in phosphorylation of FAK and Src (Figure 9C and D).

## Discussion

The cross-talk between cells and the ECM involves interactions between the individual components of the ECM and their respective receptors on the cell surface. Cell contact with ECM components initiates multiple signals that affect both cell behavior and gene expression [15]. In particular, the behavior of tumor cells is highly influenced by cell adhesion to the ECM. In our previous work, we demonstrated that tumor cells and stromal cells communicate through an amalgam of secreted soluble factors [8]. Fibroblast and prostate tumor cell cross-talk lead to fibroblast differentiation, TGF-β, and extracellular matrix down-regulation. In this study, we found that stromal fibroblast ECM significantly increased the cell adhesion, migration and organization of actin filaments in colorectal cancer cells.

Our results suggest that stromal ECM influences the amount of GAGs on tumor cells, leading to an increase in HS produced by HCT-116 cancer cells. HS proteoglycans, which are composed of a core protein and HS chains, are prevalent on the cell surface and basement membrane and have been shown to regulate various cell behaviors [21]. Syndecans are a family of transmembrane HS proteoglycans that bind ECM molecules and/or soluble ligands and transduce signals that influence cell adhesion, motility, proliferation, differentiation and morphogenesis [22-24]. The HS proteoglycan-ECM interaction is differentially regulated in cancerous versus normal cells because cancer cells are less adhesive and more migratory compared to normal cells [25].

This study showed that syndecan-2 mRNA expression in a highly metastatic colorectal cancer cell line, HCT-116, is enhanced when the cancer cells were cultured in the presence of ECM that was produced by fibroblasts. The augment of syndecan-2 was also visualized by immunoprecipitation with anti-syndecan-2 antibody. In contrast, the expression of syndecans-1 -3 and -4 decreased. Increased syndecan-2 expression has been reported in several colorectal cancer cell lines with a concomitant decrease in syndecans-1 and -4 [15,16]. Interestingly, Caco-2 cells show low syndecan-2 expression, and inhibition of syndecan-2 function in this cell line did not affect any of their adhesion, proliferation, invasion and migration [15]. Although derived from a colon (large intestine) carcinoma, when cultured under specific conditions the cells become differentiated and polarized such that their phenotype, morphologically and functionally, resembles the enterocytes lining the small intestine [26].

Syndecan-1 has been associated with tumor suppressor function, and its expression is downregulated in a variety of cancer tissues. Similarly, syndecan-4, which is mainly involved in cytoskeletal and membrane reorganization during stress fiber formation and focal adhesion in the later stages of fibroblast spreading, inhibits cell migration and tumor activity [13]. Consistent with this, the mRNA expression levels of syndecan-1 and -4 are significantly reduced in several cancer cells, including colon carcinoma cells.

On the other hand, syndecan-2 is involved in the regulation of cell adhesion in several cell lines, including epithelial cells, and several reports indicate that syndecan-2 is normally highly expressed in cells under migratory conditions [17]. These reports also indicate that syndecan-2 may function as a cell surface receptor in highly migratory tumor cells. In several colorectal cancer cell lines, syndecan-2 is highly expressed compared to normal cell lines. This increase appears to be critical for cancer cell behavior because it regulates adhesion and proliferation and, therefore, tumorigenic activity. This implies that during malignant transformation, there is a shift from the expression of anti-tumorigenic syndecans (-1 and -4) to the tumorigenic syndecan-2 [27].

HS proteoglycans are well known to interact with specific ECM proteins [28]. Fibronectin contains two binding sites for heparin, named Hep-I and Hep-II in the N- and C-terminal domains, respectively [29,30], and provides structural support for cell adhesion. Fibronectin also participates in the transduction of signals that promote actin dynamics, migration and cell proliferation. Our results showed that fibronectin is mainly responsible for the increased expression and synthesis of syndecan-2 in HCT-116 colorectal cancer cells that was induced by stromal fibroblast ECM. We also observed co-localization of syndecan-2 and fibronectin by confocal microscopy. Therefore, in addition to binding to syndecan-2, fibronectin is also able to regulate its expression.

Furthermore, blocking syndecan-2 with a specific antibody resulted in a decrease in cell adhesion and migration, as well as the decreased organization of actin filaments during cell adhesion, highlighting an important role for syndecan-2 in the regulation of actin filaments. The cytoplasmic domain of syndecan-2 interacts with Ezrin, Radixin and Moesin (ERM) family proteins, which are involved in the organization of the actin cytoskeleton and the activation of focal adhesion kinase [31-33]. Kusano and colleagues demonstrated that the induction of stress fiber formation in Lewis lung carcinoma-derived P29 cells during adhesion to fibronectin requires cooperation between integrin α5β1 with cell surface heparan sulfate from syndecan-2. Side chains with dodecasaccharide(s) enriched in the disaccharide, IdoA(2*O*S)–Glc*N*S(6*O*S), are necessary for the specific interaction between syndecan-2 and the C-terminal heparin-binding domain of fibronectin [27].

In migratory cells, forced expression of syndecan-2 induces filopodial extensions and results in rearrange of the actin cytoskeleton [34]. Our results also show that syndecan-2 is necessary for HCT-116 cells to migrate through stromal ECM. This is consistent with previous studies showing that syndecan-2 expression in colorectal cancer-derived HT-29 M6 epithelial cells induces a migratory phenotype [35] that is associated with noticeable increases in cell proliferation, invasion and anchorage-independent growth [14].

In addition to dramatic adhesion and migration changes, the contact with stromal fibroblasts ECM increased integrins alpha-2 and beta-1 expressed in HCT-116 cancer cells. Choi and colleagues showed that overexpression of syndecan-2 enhanced collagen adhesion, cell migration and invasion of normal rat intestinal epithelial cells (RIE1), and increased integrin alpha-2 expression levels. Interestingly, RIE1 cells transfected with either syndecan-2 or integrin alpha-2 showed similar adhesion and migration patterns, and a function-blocking anti-integrin alpha-2 antibody abolished syndecan-2-mediated adhesion and migration [19]. Moreover, syndecan-2 is crucial for the tumorigenic activity of sarcoma cells and this function relies on FAK-mediated signaling [36].

FAK and Src are non-receptor tyrosine kinases that control a number of cellular signaling pathways, including cell motility and survival, and playing a pivotal role in integrin-linked signal transduction, such as that associated with cell migration [37]. In several cell types, FAK directly recruits Src to the focal adhesion sites where Src potentiates activation of FAK through phosphorylation of additional tyrosine residues. Tyrosine phosphorylation of FAK and integrin molecules creates docking sites for other proteins involved in actin cytoskeleton remodeling. High levels of FAK have been found in a variety of carcinomas, including head and neck carcinomas, ovarian carcinomas, thyroid carcinomas, and colon carcinomas [36].Our findings showed that the contact of colorectal cancer cells with fibroblasts ECM resulted in increased phosphorylation of FAK and Src. Therefore, our results further reinforce the collaboration of syndecan-2 and FAK in colorectal tumorigenesis.

In summary, the cell surface heparan sulfate proteoglycan adhesion receptor, syndecan-2, plays a critical role in regulating the tumorigenic activity of HCT-116 colorectal cancer cells. It can be over-expressed by the contact of cancer cells with stromal ECM, specifically fibronectin, leading to an increase in cell adhesion and migration, probably through FAK-mediated signaling.

## Conclusion

Our results demonstrate a role for stromal ECM in regulating the expression of surface proteins in cancer cells, which are able to transduce signals that ultimately affect cell adhesion and morphology. The use of stromal fibroblast ECM alone allowed us to study its influence on the synthesis of proteoglycans and GAGs by colorectal cancer cells. Although this model does not take into consideration the cross-talk between tumor and stromal cells via soluble proteins such as growth factors, contact with ECM proteins has been shown to significantly induce alterations in cancer cell behavior. The increase of syndecan-2 expression in human colon cancer tissues has already been demonstrated [14], and our results showed that ECM proteins can be responsible for this augmentation. Therefore, although this is an *in vitro* study, we attempted to model what occurs during tumorigenesis *in vivo*, during which tumor cells are not isolated but engage in cross-talk with cancer-associated stroma.

## Methods

### Cell culture

The colorectal adenocarcinoma cell line Caco-2 and the colorectal carcinoma cell line HCT-116 were purchased from ATCC (American Type Culture Collection, Manassas, VA, USA). Human fibroblast cells primary isolated from amniotic fluid were kindly donated by Dr. Walter Pinto Júnior. These cells were properly characterized on our previous work, and the presences of fibroblasts markers, such as vimentin, were confirmed [8]. HCT-116 cells were grown in RPMI (GibcoBRL, Life Technologies Inc., Grand Island, NY), medium supplemented with 10% (v/v) fetal bovine serum (FBS, Cultilab, Campinas, Brazil), penicillin (100 units/ml) and streptomycin (100 μg/ml, Invitrogen) at 37°C in a humidified atmosphere of 5% CO_2_. Caco-2 cells and fibroblasts were grown in Dulbecco’s modified Eagle’s medium (DMEM, GibcoBRL) supplemented with 10% (v/v) FBS, penicillin (100 units/ml) and streptomycin (100 μg/ml, Invitrogen, Carlsbad, CA) at 37°C in a humidified atmosphere of 5% CO_2_.

### Preparation of stromal ECM

Fibroblasts were plated in 35-mm Petri dishes (5×10^5^ cells/ml medium, Corning Incorporated, Corning, NY) and cultured for three days. The cells were then washed with Earle’s balanced salt solution (EBSS) and incubated in PBS containing 0.025% EDTA for 20 min to detach cells while allowing the ECM to remain intact [38]. The detached cells were aspirated, and the plates were gently washed three times with EBSS and monitored by an optical microscope to ensure that all cells were removed. Colorectal cancer cells were then seeded on top of the fibroblast ECM and cultured for three days. As a control, we used cells cultured in the absence of fibroblast ECM.

### [^35^S]-Sulfate metabolic radiolabeling of GAGs

Caco-2 and HCT-116 cells were seeded on stromal fibroblast ECM and cultured for three days. Cells were then incubated with [^35^S]Na_2_SO_4_ (100 μCi/ml) for 24 h for GAG labeling. The medium was removed, and the cells were washed with PBS. The cells were then detached with 0.025% EDTA, and the ECM that was formed by these cells was extracted with 0.01% trypsin. The cells were lysed by treatment with 3.5-M urea in PBS. Proteoglycans from the medium, cell extract and matrix were precipitated for 18 h at −20°C with 3 volumes of ethanol in the presence of heparan sulfate as a carrier. Ethanol pellets were dried and subjected to proteolysis with maxatase, a protease from *Sporobacillus*, (4-mg/ml) in 0.05-M Tris–HCl, pH 8.0 for 24 h at 60°C. Labeled GAGs were dried and resuspended in 100 μl of water. The composition of GAGs in each compartment (medium, cell extract and matrix) was analyzed by agarose gel electrophoresis.

### GAG analysis by agarose gel electrophoresis

Samples (5 μl) were applied to agarose gel slabs in 0.05-M 1,3-diaminepropane acetate buffer pH 9.0 [39,40]. Following electrophoresis, GAGs were precipitated for 1 h with 0.2% cetyltrimethylammonium bromide. ^35^S-radiolabeled GAGs were visualized after exposure to Cyclone™ Storage Phosphor Screen (Packard Instrument Company Inc., Japan) for 24 h, and images were acquired using a Cyclone™ Storage Phosphor System (Packard Instrument Company Inc., Japan). CS and HS were identified after agarose gel electrophoresis, based on the difference in migration under this electrophoresis condition, as previously described [41,42]. Quantitative analysis was performed by densitometry using the Opti-Quanti Software.

### RNA extraction and real time PCR

Total RNA was extracted from cultured cells using Trizol® reagent (Invitrogen) according to the manufacturer’s guidelines. The total RNA extracted was used as the template for the reverse transcriptase reaction. Aliquots of cDNA were amplified using the following primers: human syndecan-1 (NM_001006946) forward (F): AGGGCTCCTGCACTTACTTGCTTA and reverse (R): ATGTGCAGTCATACACTCCAGGCA; human syndecan-2 (NM_002998) F: AACTTCTGCCGTAGCTCCCTTTCA and R: AGGCTGCTCTCTGAAGCTCTTCTT; human syndecan-3 (NM_014654) F: AAGGAGGTGCTCGTAGCTGTGATT and R: TCCTGCTTGTCAGGCTTCTGGTAT; human syndecan-4 (NM_002999) F: CCAGTTTGATGTTGCTGGGTGGTT and R: AGCCCTAGAGCCTGAAGAAAGCAA; human β-actin (NM_001101) F: ACCAACTGGGACGACATGGAGAAA and R: TAGCACAGCCTGGATAGCAACGTA. After an initial denaturation step at 94°C for 5 min, the following steps were carried out for 35 cycles: denaturation at 94°C for 30 s, annealing at 55°C for 30 s and extension at 72°C for 60 s. The PCR products were analyzed on 1% agarose gels and sequenced to confirm their identities. Quantitative real-time PCR was performed using SYBR^®^ Green PCR Master Mix and AmpliTaq-GOLD polymerase (Applied Biosystems, Foster City, CA). Reactions were performed on the ABI PRISM 7500 Real Time PCR System (Applied Biosystems). Briefly, 1 μl of diluted cDNA was added to SYBR^®^ Green PCR Master Mix, and forward and reverse primers were added to a final concentration of 0.5 μM. All reactions were performed in triplicate. PCR cycling conditions were as follows: DNA denaturation for 10 min at 95°C and then 35 cycles of 95°C for 15 s, 60°C for 1 min and 72°C for 30 s. Fluorescence data were recorded at the end of each 72°C step. A DNA melting profile was subsequently run from 72°C to 95°C with a ramp of 1°C/5 s. Fluorescence data were recorded continuously during the melt profile. The relative expression levels of genes were calculated using the 2^-ΔΔCT^ method [43]. The levels of target gene expression in each sample were normalized to the average expression level of the endogenous control, β-actin.

### Immunoprecipitation of syndecan-2

Proteoglycans synthesized by the cells were metabolically labeled with [^35^S]Na_2_SO_4_ (100 μCi/ml), as described above. At the end of the incubation period, the culture medium was removed and the cells were washed twice with PBS. The cells were scrapped from the dish with a lysis buffer containing 1% NP-40, 10% glycerol, 135 mM NaCl, 20 mM Tris–HCl pH 8.0. Cell lysates containing equal amounts of total protein were incubated with anti-syndecan-2 (1:50, Santa Cruz Biotechnology, Santa Cruz, CA) at 4°C for 2 hours. The lysates were then incubated with 50 μl of a suspension of protein A-sepharose beads in PBS (1:1, wt/v) at 4°C for 1 h. The beads were pelleted by centrifugation, washed with 3 × 1 ml of the lysis buffer, 2 × 1 ml of 20 mM Tris–HCl pH 8.0 containing 1 M LiCl and 2 × 1 ml of 20 mM Tris–HCl pH 8.0. All solutions contained protease inhibitors (1 mM phenylmethylsulfonyl fluoride, 10 mM N-ethylmaleimide, 10 mM EDTA). The proteoglycans were identified by agarose gel electrophoresis as previously described [41,42].

### Protein preparation and western blotting

For the extraction of proteins, HCT-116 cells were seeded on stromal fibroblast ECM and cultured for three days. Adhered cells were removed from the culture dishes with the aid of a cell scrapper in cell lysis buffer (Cell Signaling, MA, USA) containing proteinase inhibitor cocktail (Roche, Mannheim, Germany) and, thereafter, exposed to 10 freeze/thaw cycles. For the extraction of proteins from fibroblasts ECM, the cells were detached with 0.025% EDTA, and the remaining ECM was removed from the culture dishes with the aid of a cell scrapper. The total protein content of the cell extracts was measured using the Micro BCA Protein Assay Kit (Pierce, Rockford, IL). The lysate proteins were separated according to molecular mass by sodium dodecyl sulfate polyacrylamide gel electrophoresis (SDS-PAGE) using a 10% polyacrylamide gel (Merck, Whitehouse Station, NJ) and transferred to polyvinylidene difluoride membranes (Millipore, Bedford, MA, USA) by 2.5 h electroblotting at 200 mA constant current in blotting buffer (20 mM Tris-base, 150 mM glycine, 20% methanol) using a Mini Trans-Blot Electrophoretic Transfer Cell (Bio-Rad Laboratories). The membranes were quenched for 1 h with 5% non-fat dry milk in TBST buffer (200 mM Tris/HCl buffer, pH 8.0 containing 150 mM NaCl and 0.05% Tween-20) and then incubated overnight at 4°C with primary antibodies: rabbit anti-syndecan- 2 (Santa Cruz), goat polyclonal anti-fibronectin (Santa Cruz), goat anti-laminin (Santa Cruz),rabbit anti-collagen-I (Pierce), rabbit anti-phospho-Src (Y416) (Cell Signaling), rabbit anti-Src (Cell Signaling), rabbit anti-phospho-FAK (Y397) (Cell Signaling), rabbit anti-FAK (Cell Signaling), mouse anti-integrin β1 (Santa Cruz), goat anti-integrin α2 (Santa Cruz) and anti-β-actin (Sigma Chemical Company, St. Louis, MO), diluted in 1% bovine serum albumin (BSA) in TBST. Thereafter, the membranes were further incubated for 1 h at room temperature with the appropriate secondary antibodies conjugated with Horseradish peroxidase (Cell Signaling) diluted in 1% BSA in TBST. After each step, membranes were sequentially washed three times with TBST. Chemiluminescence signal detection was performed using the gel documentation system G:Box Chemi HR16 (Syngene, Frederick, MD, USA). Densitometric analysis was performed using the Scion Imaging software (Scion Corporation), using β-actin as a control for each sample.

### Flow cytometry

HCT-116 cells were seeded on top of stromal fibroblast ECM, fibronectin, laminin or collagen type I and detached with 0.025% EDTA in PBS. For each sample, 10^6^ cells were used. The cells were washed with PBS and fixed with 2% paraformaldehyde in PBS for 30 min. Staining was performed by incubating cells with primary antibody against the extracellular domain of human syndecan-2 (1:100 dilution; Santa Cruz Biotechnology) for 2 h, followed by incubation with anti-IgG conjugated to Alexa 488 (1:300 dilution, Invitrogen) for 40 min. Data were collected using the FACSCalibur flow cytometer (Becton Dickinson, CA, USA).

### Immunocytochemistry

For immunocytochemical staining, cells were washed with PBS, fixed with paraformaldehyde for 30 min and blocked with 5% FBS. The cells were then incubated for 2 h with the following primary antibodies: rabbit polyclonal anti-human syndecan-2 (1:100 Santa Cruz Biotechnology) or goat polyclonal anti-fibronectin (1:100 Santa Cruz Biotechnology). After washing with PBS, the sections were incubated at room temperature for 1 h with the appropriate secondary antibody: anti-rabbit IgG or anti-goat IgG, all produced in donkeys and conjugated to Alexa 594 or Alexa 488 (1:200 Invitrogen). The cells were then counterstained with a second set of primary and secondary antibodies. Nuclei were stained using DAPI (0.5 μg/ml, Molecular Probes, Carlsbad, CA). The cells were mounted using Fluoromount G (2:1 in PBS, Electron Microscopy Sciences, Hatfield, PA) and analyzed using a Zeiss LSM510 scanning confocal inverted microscope (Jena, Germany). Co-localization images were generated using ImageJ software (http://rsb.info.nih.gov/ij/) or Zeiss LSM Image Browser.

### Cell adhesion assay

Twenty-four-well plates were coated with stromal fibroblast ECM as described above. HCT-116 cells, previously grown on top of fibroblast ECM for three days, were detached with 0.02% trypsin-0.2-mM EDTA, centrifuged and pre-incubated with anti-syndecan-2 (1:100 Santa Cruz Biotechnology), human IgG (1:100 Jackson ImmunoResearch) or no antibodies for 1 h at 37°C in a 5% CO_2_ atmosphere. The cells were then seeded on stromal ECM at a concentration of 2×10^5^ cells/ml medium and cultured for different lengths of time. Actin filaments were stained with Alexa 488-conjugated phalloidin and visualized under a fluorescence microscope. The number of adherent cells was measured by the MTT colorimetric assay. Medium (200 μl) containing 0.5-mg/ml MTT (Sigma) was added to each plate, and cells were incubated for 2 h. The medium was then removed, and 200 μl of dimethyl sulfoxide was added to each well for 30 min at room temperature. The mean absorbance at 540 nm in each set of samples was measured in duplicate using a 96-well microtiter plate reader.

### Cell migration assay

Transwell membranes (Costar, Corning, 8-μm pore size) were coated with stromal fibroblast ECM as described above. HCT-116 cells, previously grown on top of fibroblast ECM for three days, were detached with 0.02% trypsin-0.2-mM EDTA, centrifuged and pre-incubated with anti-syndecan-2 (1:100 Santa Cruz Biotechnology), human IgG (1:100 Jackson ImmunoResearch) or no antibodies for 1 h at 37°C in a 5% CO_2_ atmosphere. 2×10^4^ HCT-116 cells were plated in 200 μl of RPMI medium in the top of chamber. In the bottom chamber, 500 μl of RPMI supplemented with 10% FBS was added. Migration assays were carried out for 24 h in the tissue culture incubator. The cells were fixed by replacing the culture medium in both bottom and top chambers with 4% formaldehyde dissolved in PBS. After fixation for 15 min at room temperature, the chambers were rinsed in PBS and stained with 0.2% crystal violet for 10 min. After the chambers were washed five times by dipping in a large beaker of dH_2_O, cells (now blue in color) in the upper chamber were removed with several Q-tips. Remaining cells were counted using an inverted microscope equipped with a 10X objective and plotted as the percentage of migratory cells.

### Statistical analysis

Statistical significance was determined by paired t test on Microsoft Excel (Redmond, WA, USA). All experiments were performed in triplicate unless stated otherwise. Reproducible results were obtained and representative data are shown. p-values < 0.05 were considered significant.

## Abbreviations

ECM: extracellular matrix; HS: heparan sulfate; CS: chondroitin sulfate; GAGs: glycosaminoglycans; FBS: fetal bovine serum; DMEM: Dulbecco’s modified Eagle’s medium; EBSS: Earle’s balanced salt solution; PBS: phosphate buffered saline; EDTA: ethylenediaminetetraacetic acid; MTT: 3-(4,5-Dimethyl-2-thiazolyl)-2,5-diphenyl-2H-tetrazolium bromide; FAK: Focal Adhesion Kinase; SFK: Src Family Kinases; DMSO: dimethyl sulfoxide; DAPI: 4,6-diamidino-2-phenylindole.

## Competing interests

The authors declare that they have no competing interests.

## Authors’ contributions

CMV performed all experiments and prepared the manuscript. CMV and RR designed the experiment. HBN contributed scientific support. LT is responsible for the entire study. All the authors have read and approved the final manuscript.
